# Врожденный гипопитуитаризм при делециях 18 хромосомы

**DOI:** 10.14341/probl12761

**Published:** 2021-07-13

**Authors:** А. В. Болмасова, М. А. Меликян, З. Ш. Гаджиева, А. А. Пучкова, А. В. Дегтярева, В. А. Петеркова

**Affiliations:** Национальный медицинский исследовательский центр эндокринологии; Национальный медицинский исследовательский центр акушерства, гинекологии и перинатологии им. акад. В.И. Кулакова; Национальный медицинский исследовательский центр эндокринологии; Национальный медицинский исследовательский центр эндокринологии; Национальный медицинский исследовательский центр акушерства, гинекологии и перинатологии им. акад. В.И. Кулакова; Национальный медицинский исследовательский центр акушерства, гинекологии и перинатологии им. акад. В.И. Кулакова; Первый Московский государственный медицинский университет им. И.М. Сеченова (Сеченовский университет); Национальный медицинский исследовательский центр эндокринологии

**Keywords:** врожденный гипопитуитаризм, синдром De Grouchy, моносомия 18p-, моносомия 18q-, гипогликемия, холестаз

## Abstract

Врожденный гипопитуитаризм — редкое заболевание, причиной которого могут быть изолированные пороки развития хиазмально-селлярной области, мутации генов, участвующих в развитии гипофиза (например, PROP1, PIT1), и хромосомные нарушения.Делеции 18 хромосомы (синдром De Grouchy 1 и 2 типов) — группа редких генетических заболеваний с частотой встречаемости 1:50 000. Гипопитуитаризм при данном синдроме выявляется в 13–56% случаев и зависит от размера и локализации делеции.В статье описана серия клинических случаев врожденного гипопитуитаризма при делециях короткого и длинного плеч 18 хромосомы.Все дети имели характерные стигмы дизэмбриогенеза и задержку психоречевого развития различной степени выраженности. Обращало на себя внимание наличие мышечной гипотонии, дисфагии, дыхательных нарушений в раннем неонатальном периоде. У пациентов отмечалось наличие различных врожденных пороков развития в сочетании с гипопитуитаризмом, проявления которого варьировали от изолированного СТГ-дефицита до множественных тропных недостаточностей аденогипофиза. Особенностью течения гипопитуитаризма в период новорожденности являлось наличие рецидивирующих гипогликемий в сочетании с синдромом холестаза, которые быстро купировались на фоне заместительной гормональной терапии.Двум пациентам проводился хромосомный микроматричный анализ, при помощи которого были определены точная локализация области делеции и гены, выпавшие при данном дефекте, что позволило оптимизировать тактику дальнейшего ведения.

## Abstract (en)

Congenital hypopituitarism is a rare disease. It can be caused by isolated inborn defects of the pituitary, gene mutations (PROP1, PIT1), and chromosomal abnormalities.Deletions of chromosome 18 (De Grouchy syndrome types 1 and 2) are a group of rare genetic diseases with a frequency of 1:50,000. Hypopituitarism in these syndromes is detected in from 13 to 56% of cases and depends on the size and location of the deleted segment.We have described a series of clinical cases of patients with congenital hypopituitarism due to deletions in chromosome 18. All children had a characteristic dysmorphic features and delayed mental and speech development. Within first months of life, patients developed muscular hypotension, dysphagia, and respiratory disorders. The patients had various congenital malformations in combination with hypopituitarism (isolated growth hormone deficiency and multiple pituitaryhormone deficiencies). In the neonatal period, there were the presence of hypoglycemia in combination with cholestasis.Hormone replacement therapy led to rapid relief of symptoms.Сhromosomal microarray analysis in 2 patients allowed us to identify exact location of deleted area and deleted genes and optimize further management for them.

## АКТУАЛЬНОСТЬ

Делеции 18 хромосомы (моносомия 18p-, синдром 18p-, частичная моносомия 18p-, моносомия 18q-, синдром 18q-) представляют собой группу редких генетических заболеваний с частотой встречаемости около 1:50 000. Впервые фенотип пациентов с делецией короткого плеча был описан в 1963 г. французским генетиком Jean de Grouchy [1]. В дальнейшем были выделены синдром De Grouchy 1 типа (делеция короткого плеча 18 хромосомы) и синдром De Grouchy 2 типа (делеция длинного плеча 18 хромосомы). В обоих случаях в структуре синдрома может отмечаться врожденный гипопитуитаризм (от 14 до 56%), который часто ассоциирован с другими врожденными патологиями и сопутствующими заболеваниями [2][3].

При моносомии 18p- (OMIM #146390) в большинстве случаев делеции происходят de novo (89%) и чаще встречаются у девочек (2/3). Чаще всего моносомия короткого плеча происходит по материнской хромосоме, и в 50% — в центромерной области [4]. Фенотипические проявления у пациентов включают задержку умственного развития (средний IQ=69 баллов), задержку роста, задержку речевого развития, голопрозэнцефалические пороки развития, птоз и фенотипические особенности (плоская спинка носа, широкий рот с короткой верхней губой, микрогнатия, оттопыренные уши, короткая шея с крыловидными складками, кифосколиоз). Гипопитуитаризм при синдроме 18p- встречается в 13% случаев и относится к редким проявлениям синдрома, как и аутоиммунные заболевания, алопеция и мышечная дистония. У пациентов с микроделециями фенотип вариабелен и зависит от размера и локализации делеции. При утрате целого плеча с захватом центромерной области фенотип менее гетерогенный и включает наиболее полный спектр нарушений [5].

При моносомии 18q- (OMIM #601808) фенотип крайне вариабелен. Выделяют 2 вида делеций: проксимальный (18q11.2-q21.1) и дистальный (18q21.1-q23) типы.

Дистальный тип делеций характеризуется задержкой речевого и умственного развития, врожденными пороками сердца, патологией ЦНС, ортопедическими нарушениями, офтальмологическими проблемами (косоглазие, нистагм, миопия), дефицитом IgA, гипотиреозом, тугоухостью, патологией почек. Гипопитуитаризм (изолированный дефицит гормона роста) является достаточно частым состоянием для данного типа хромосомных нарушений [6–9].

Для проксимального типа делеций, помимо задержки умственного и речевого развития, характерны гипоплазия мозолистого тела, гидронефроз почек, пороки сердца, частые синуситы, косоглазие, экзема, обструктивное апноэ, кондуктивная тугоухость. Дефицит гормона роста при проксимальной делеции встречается реже (до 14% случаев).

Учитывая, что фенотипические проявления моносомии 18q- крайне гетерогенны и зависят от размера, локализации делеции и дозозависимости генов, попавших в область делеции, невозможно описать четкий фенотип, так как он варьирует от пациента к пациенту [9].

К общим чертам можно отнести: гипоплазию средней части лица, короткие и наклоненные вниз или вверх глазные щели, эпикант, низко посаженные уши с выступающим противозавитком (anthelix).

Для всех детей с моносомией короткого и длинного плеч 18 хромосомы характерно наличие в неонатальном периоде мышечной гипотонии, дисфагии, дыхательных нарушений (респираторного дистресс-синдрома). Гипогликемия и холестаз в период новорожденности могут быть одними из проявлений врожденного гипопитуитаризма и при несвоевременной диагностике и терапии приводить к жизнеугрожающим состояниям.

## ОПИСАНИЕ СЛУЧАЕВ

Нами было обследовано 4 ребенка с патологией 18 хромосомы (3 ребенка с моносомией 18p- и 1 ребенок с моносомией 18q-), одним из проявлений которой был врожденный гипопитуитаризм.

Всем детям диагноз был подтвержден молекулярно-генетически (2 пациентам проводилось только кариотипирование, 2 детям — хромосомный микроматричный анализ).

Врожденный гипопитуитаризм был установлен на основании гормонального обследования и характерных клинических проявлений. Все дети получали заместительную гормональную терапию под контролем эндокринолога, а также наблюдались у смежных специалистов в зависимости от сопутствующей патологии.

Данные по развитию речи и приобретению навыков были получены путем анкетирования законных представителей.

Результаты физикального, лабораторного и инструментального исследования

Пациент 1

Доношенная девочка родилась от неродственного брака, 5-й беременности (1, 2-я беременности — здоровые дети, 3-я — медицинский аборт, 4-я — здоровый ребенок). Антропометрические показатели при рождении соответствовали сроку гестации (табл. 1), оценка по шкале Апгар составила 6/8 баллов. При рождении у ребенка отмечались фенотипические особенности: широкий рот, низко расположенные ушные раковины, плоская переносица (рис. 1а). В конце первых суток жизни отмечался эпизод апноэ на фоне гипогликемии 0,7 ммоль/л, купированный внутривенным введением раствора глюкозы. В течение первых 10 дней были отмечены нарастание гипербилирубинемии за счет прямой фракции (общий билирубин — 360 мкМ/л, прямой — 46 мкМ/л), умеренный синдром цитолиза (аспартатаминотрансфераза (АСТ) — 55 ед/л, аланинаминотрансфераза (АЛТ) — 35 Ед/л), гиперлипидемия, гипохолия стула при отрицательных маркерах воспаления и отсутствии признаков, свидетельствующих об инфекционной патологии. Исключены некоторые наследственные болезни обмена (тирозинемия и галактоземия). У ребенка была заподозрена атрезия желчевыводящих потоков и проведена краевая биопсия печени, по результатам которой выявлены признаки хронического перипортального гепатита низкой степени гистологической активности. Данных за атрезию желчевыводящих протоков не получено. По результатам гепатобилиарной сцинтиграфии было выявлено снижение выделительной функции гепатоцитов, данных за атрезию желчевыводящих протоков также не получено. В ходе дальнейшего обследования у ребенка были выявлены врожденный порок сердца (дефект межжелудочковой перегородки) и врожденная катаракта. Учитывая наличие особенностей фенотипа, множественных пороков развития, было проведено кариотипирование, по результатам которого диагностирована моносомия по короткому плечу 18 хромосомы (46ХХ,del (18)(p11.1; p11.32)). Обращало на себя внимание сохранение эпизодов гипогликемии до 2,6 ммоль/л на фоне удлинения голодных промежутков.

**Table:** Таблица 1. Клинико-лабораторные данные пациентов

Показатель	Пациент 1	Пациент 2	Пациент 3	Пациент 4
Пол	ж	м	м	ж
Возраст на момент публикации	9 лет 4 мес	5 лет 3 мес	2 года 10 мес	3 года 7 мес
Кариотип	46 ХХ, del (18)(p 11.1; p 11.32)	46 ХY, del (18)(p11.32-p11.21)	46XY,del(18)(p11.2)	46ХХ,del(18q22-q23)
Микроматричный хромосомный анализ	Нет	Нет	Молекулярный кариотип: arr[hg38] 18p11.31p11.21(3067756_14489102)x1Гены, расположенные в районе дисбаланса:AFG3L2, APCDD1 GNAL, LAMA1, MC2R, NDUFV2, PIEZO2, TGIF1, TUBB6	Молекулярный кариотип: arr[hg19]18q22.3q23(69283126_78014123)х1Гены, расположенные в районе дисбаланса: CBLN2, NETO1,FBXO15, TIMM21, CYB5A, FAM69C, CNDP1, CNDP2, ZNF407, TSHZ1, ZNF516, ZNF236, MBP, GALR1, SALL3, ATP9B,NFATC1, CTD1, KCNG2, TXNL4A, PARD6G
Масса тела при рождении, г	3210	2700	2300	3240
SDS массы тела	-0,18	-1,69	-1,68	0,34
Длина тела при рождении, см	50	48	44	50
SDS длины тела	0,37	-1,09	-2,22	0,68
Оценка по шкале Апгар, баллы	6/8	7/8	6/7	8/9
Мышечная гипотония	+	+	+	+
Неонатальный холестаз	+	-	-	Гипербилирубинемия
Гипогликемия	+	+	-	-
Дыхательные нарушения в раннем неонатальном периоде	+	-	+	+
Проблемы с кормлением	+	+	-	+
Задержка моторного развития	+	+	+	+
Задержка речевого развития	+	+	+	+
Задержка умственного развития	+	+	+	+
СТГ-дефицит	+	*	+	*
Вторичный гипотиреоз	+	+	-	-
Вторичный гипокортицизм	+	+	-	-
МРТ-данные	МР-картина «частично пустого турецкого седла»	Агенезия мозолистого тела и эктопия нейрогипофиза	Гипоплазии аденогипофиза, воронки, эктопия нейрогипофиза	Не проводилось
Врожденный порок сердца	Дефект межжелудочковой перегородки	-	-	-
Врожденная катаракта	+	-	-	-
Тугоухость (кондуктивная)	-	-	-	+
Птоз/полуптоз	+	+	+	-
Единственный центральный резец верхней челюсти	-	-	+	-
Нарушение походки (атаксия)	+	-	_	-
Гипоплазия зрительных нервов	-	+	-	-

В возрасте 3,5 мес ребенок впервые был осмотрен эндокринологом. При обследовании отмечались умеренная гепатомегалия, прямая гипербилирубинемия (общий билирубин — 76,32 пмоль/л, прямой билирубин — 23,18 мкмоль/л), синдром цитолиза (АСТ — 214,4 Ед/л, АЛТ — 83,5 Ед/л), гипогликемия до 1,1 ммоль/л на фоне продленного голодного промежутка (4,5 ч).

**Figure:**
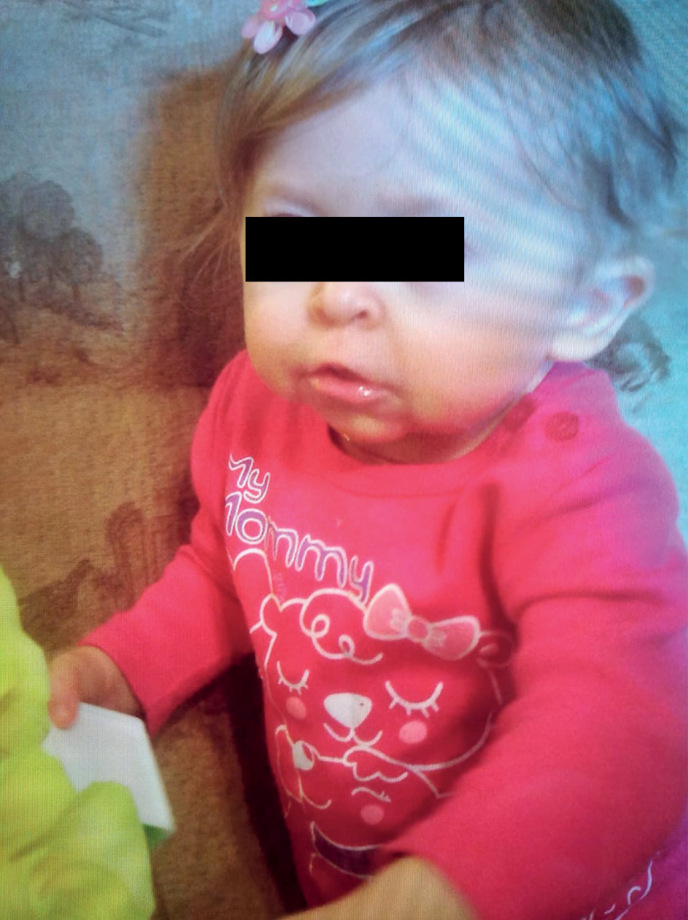
Рисунок 1а. Фото пациента 1 в возрасте 3,5 года.

По данным гормонального обследования был подтвержден гипопитуитаризм: вторичный гипотиреоз, вторичный гипокортицизм (табл. 2). Ребенку была назначена заместительная гормональная терапия левотироксином натрия (12,5 мкг/сут) и гидрокортизоном (1 мг/кг/сут). По данным МРТ головного мозга были выявлены признаки «частично пустого турецкого седла». Синдром холестаза и цитолиза, а также эпизоды гипогликемии купировались на фоне заместительной терапии в течение нескольких недель. В возрасте 2 лет в связи с выраженной задержкой роста (SDS =-8,2) ребенку было проведено дополнительное обследование: костный возраст соответствовал 8 мес, инсулиноподобный фактор роста (ИФР-1) — 32 нг/мл, что подтвердило наличие дефицита гормона роста. На фоне заместительной терапии гормоном роста (0,033 мг/кг/сут) отмечалась выраженная положительная динамика (рис. 2). В настоящий момент пациентке 9 лет (рис. 1б). Девочка получает заместительную гормональную терапию (гидрокортизон — 12,5 мг/сут = 15,2 мг/м2/сут; левотироксин натрия — 50 мкг/сут; рекомбинантный гормон роста — 0,033 мг/кг/сут). Сохраняется задержка роста. В психомоторном развитии у ребенка отмечается выраженная задержка. Самостоятельно ходит с 5 лет, также отмечается грубая задержка речевого развития (говорит короткими предложениями с применением жестов с 6 лет) (табл. 3, рис. 2). Сохраняются трудности с кормлением (поперхивается при приеме твердой пищи, плохо жует). Девочка неоднократно проходила курсы реабилитации, наблюдается логопедом, дефектологом.

**Figure:**
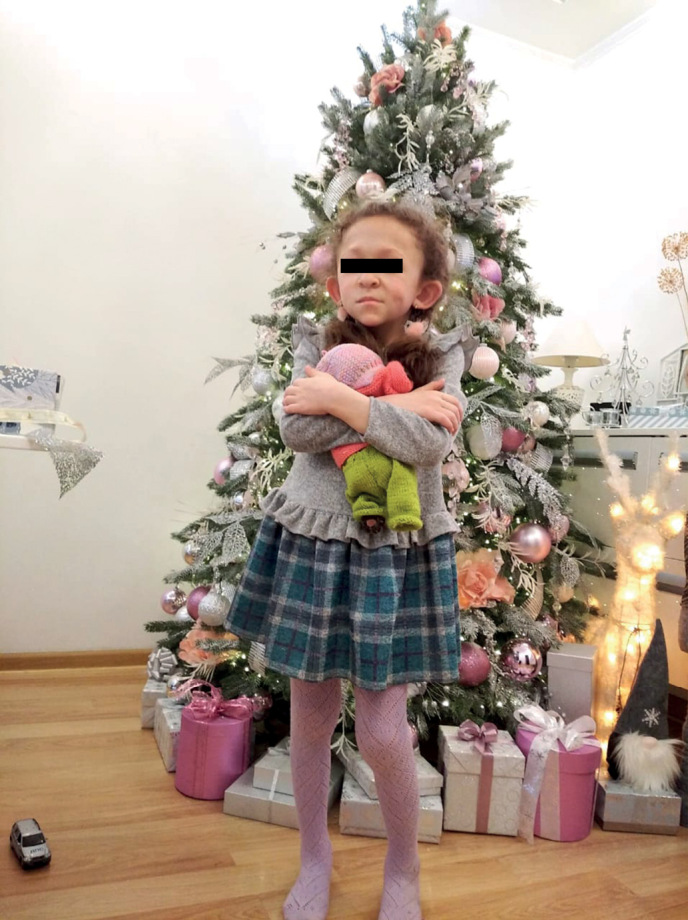
Рисунок 1б. Фото пациента 1 в возрасте 9 лет.

**Figure:**
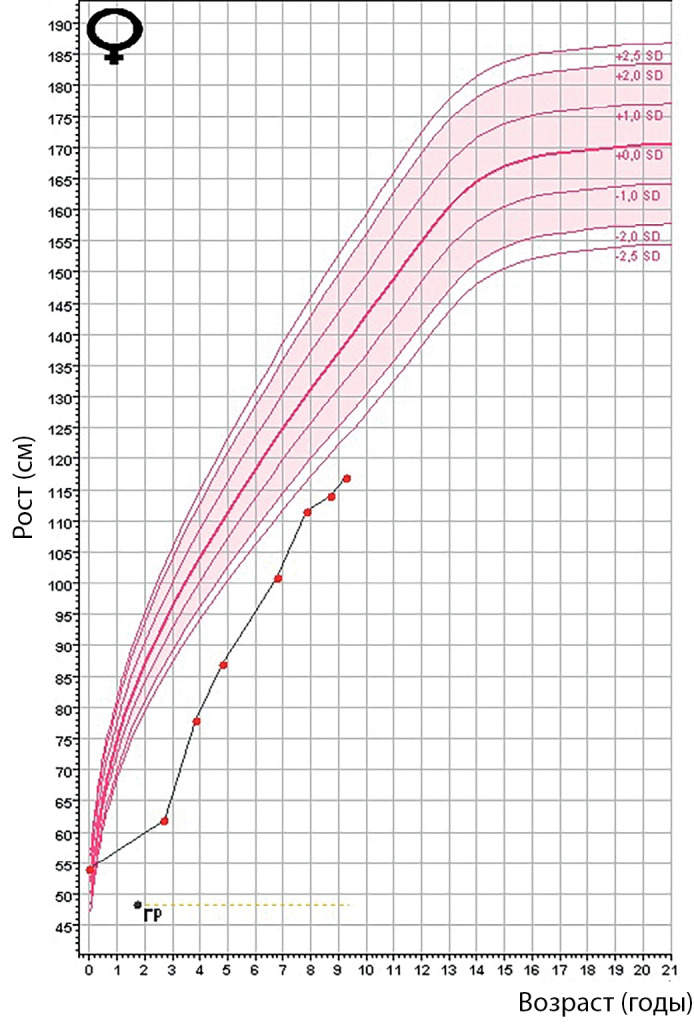
Рисунок 2. График роста пациента 1.

**Table:** Таблица 2. Данные гормональных обследований до начала гормональной терапии

Показатель	Пациент 1	Пациент 2	Пациент 3	Пациент 4
Возраст исследования, месяцы	3,5	3	24	8 /36
АКТГ, пг/млреференс: 7,2–63,3	6,05	8	25,03	не исследовался
Кортизол, нмоль/лреференс: 77–630	<11	28,9	435,2	277
ТТГ, мМед/лреференс: 0,64–5,76	1,6	3,28	1,42	1,9
свТ4, пмоль/лреференс: 11,5–20,4	7,37	8,4	12,56	13,28
ИФР-1, нг/мл	32*	48	13,43	59,9/49,8
SDS ИФР-1референс: +/-2	-3,2	-0,94	-5,37	-0,41/-1,46

**Table:** Таблица 3. Психомоторное развитие пациентов. Возраст приобретения основных навыков (результаты анкетирования)

Показатель	Пациент 1	Пациент 2	Пациент 3	Пациент 4
Возраст ребенка на момент опроса	9 лет 4 мес	5 лет 3 мес	2 года 10 мес	3 года 7 мес
Держит голову	С 1 года	С 3 мес	С 8 мес	С 4 мес
Переворачивается	С 3 лет	С 6 мес	С 5 мес	С 5,5 мес
Ползает	Не ползала	С 10 мес	С 11 мес	Не ползала
Стоит без опоры	С 4 лет	С 1 года	С 1,5 лет	С 1 года 7 мес
Ходит без поддержки	С 5 лет	С 1 года 10 мес	С 1,5 лет	С 1 года 9 мес
Произносит отдельные слова	С 3 лет	С 3,5 лет	Речь отсутствует	С 3 лет
Выполняет простые просьбы(понимает обращенную речь)	С 2 лет	С 3 лет	С 2 лет	С 1 года
Говорит предложениями	С 6 лет	С 4 лет (скандированная речь, заученные предложения)	Речь отсутствует	С 3 лет 5 мес
Пользуется горшком/туалетом днем	С 5 лет	Не всегда	Нет	С 2 лет 7 мес
Пользуется горшком/туалетом ночью	Нет	Нет	Нет	С 2 лет 7 мес
Посещает детский сад	Коррекционный с 6 лет (с мамой)	Нет, ежедневные индивидуальные занятия, введение в малые группы детей	Нет	Нет, посещает занятия с логопедом, занятия с сенсорной интеграцией
Посещает школу (общеобразовательную/коррекционную — указать)	С 8 лет (надомное обучение)	Нет	Нет	Нет

Пациент 2

Доношенный мальчик родился от матери, страдающей нейрофиброматозом 2 типа. При рождении антропометрические показатели соответствовали нижней границе нормы, оценка по шкале Апгар 7/8 баллов (см. табл. 1). При осмотре отмечались стигмы дизэмбриогенеза: «оттопыренные», низко посаженные уши, широкий рот, гипертелоризм сосков, врожденный полуптоз (рис. 3). С первых суток жизни отмечались рецидивирующие гипогликемии до 0,9 ммоль/л, в связи с чем проводилась инфузионная терапия раствором глюкозы. В 3 нед жизни было проведено исследование, по данным которого исключен гиперинсулинизм, установлен диагноз: Гипопитуитаризм, вторичный гипотиреоз, вторичный гипокортицизм (табл. 2). Была инициирована терапия гидрокортизоном (1 мг/кг/сут), на фоне которой гипогликемии купировались, далее назначен левотироксин натрия (12,5 мкг/сут). Мальчику также дважды исследован уровень ИФР-1 в возрасте 2 и 3 мес — показатели в пределах нормы (49–54 нг/мл).

В возрасте 3 мес ребенок был осмотрен окулистом, выявлена гипоплазия зрительных нервов. Проведена магнитно-резонансная томография (МРТ) головного мозга, по данным которой диагностирована агенезия мозолистого тела и эктопия нейрогипофиза. Была заподозрена септо-оптическая дисплазия. Проведен молекулярно-генетический анализ («Панель гипопитуитаризм»), мутаций выявлено не было. Далее мальчик регулярно наблюдался эндокринологом, несмотря на увеличение дозы глюкокортикоидов (дозировка по гидрокортизону 17,7 мг/м2/сут) сохранялись гипогликемии на фоне удлинения голодного промежутка.

В возрасте 6 мес, учитывая наличие гипопитуитаризма, рецидивирующих гипогликемий, сохраняющихся на фоне высоких доз гидрокортизона, несмотря на нормальные темпы роста, ребенку по решению консилиума была назначена заместительная терапия рекомбинантным гормоном роста в дозировке 0,028 мг/кг/сут (рис. 4) ввиду высокой вероятности дефицита соматотропного гормона (СТГ). На фоне терапии гипогликемии купировались. Стоит отметить, что в течение первых 3 лет жизни у ребенка отмечались частые кризы надпочечниковой недостаточности на фоне респираторных заболеваний, сопровождающиеся рвотами и гипогликемией.

Учитывая наличие стигм дизэмбриогенеза, было проведено кариотипирование, по данным которого выявлена делеция короткого плеча 18 хромосомы (46 ХY, del (18)p11.32-p11.21). В психомоторном развитии отмечалась выраженная задержка (ходит с 1 года 10 мес), а также задержка речи (в 3,5 года повторял отдельные слоги, обращенную речь понимал частично). В динамике мальчику проведена МРТ головного мозга — выявлены множественные очаги в субкортикальном белом веществе обоих полушарий, предположительно невриномы/очаги демиелинизации. Учитывая отягощенный наследственный анамнез, ребенку был проведен молекулярно-генетический анализ и выявлена мутация гена NF2, подтверждающая наличие нейрофиброматоза 2 типа. Учитывая риск развития объемных образований ЦНС, доза гормона роста была снижена с 0,028 до 0,025 мг/кг/сут. На момент написания статьи ребенку 5 лет, отмечается значимый прогресс речевого и психомоторного развития на фоне занятий с логопедом, дефектологом (см. табл. 3). На фоне заместительной терапии гормона роста отмечаются удовлетворительные темпы роста (рис. 4).

**Figure:**
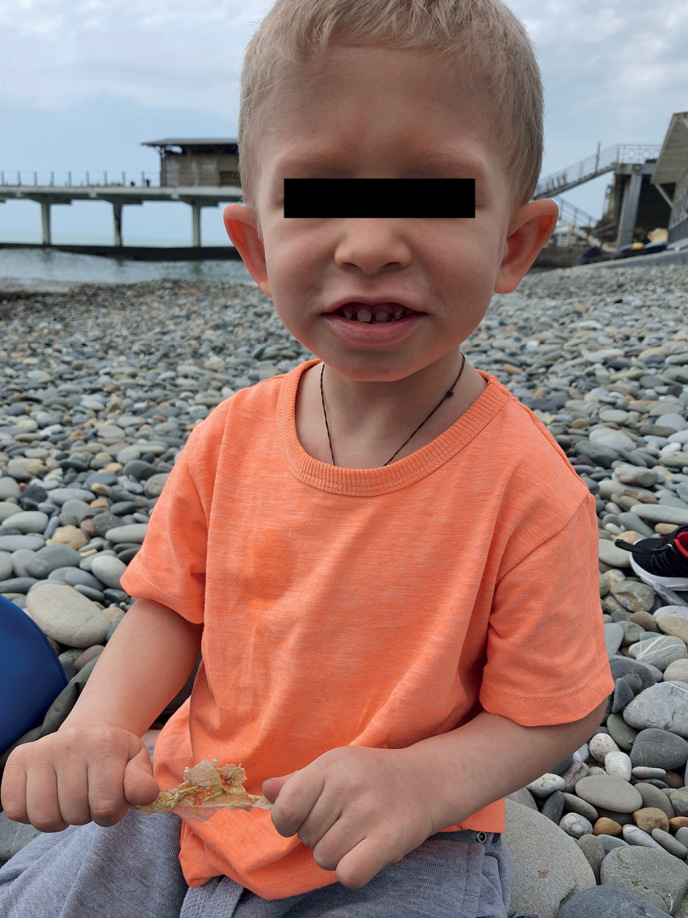
Рисунок 3. Фото пациента 2 в возрасте 4,5 года.

**Figure:**
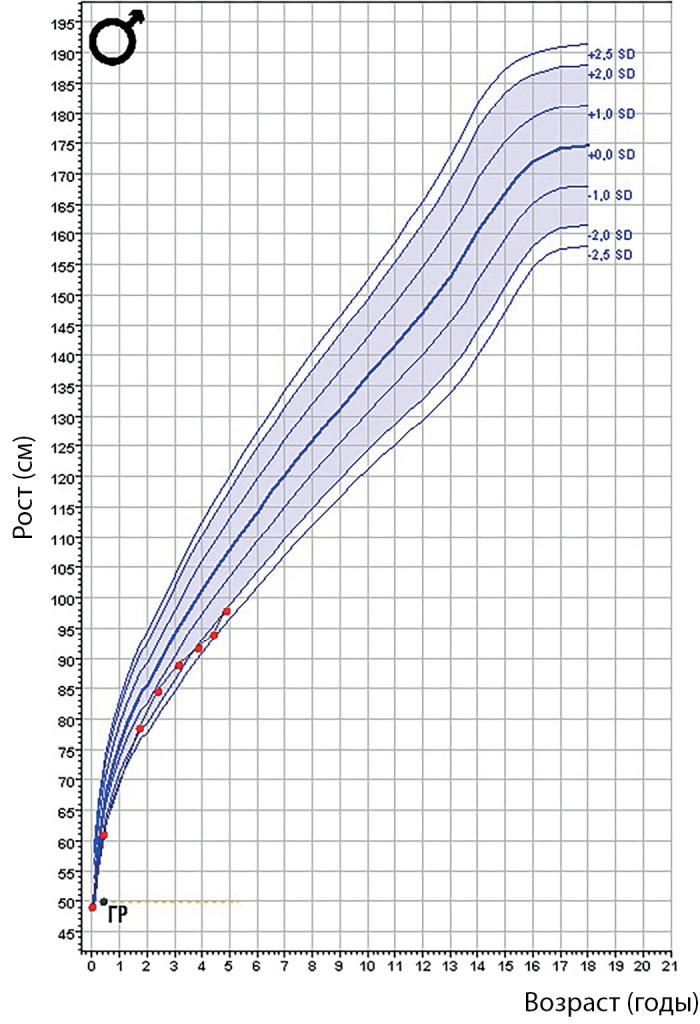
Рисунок 4. График роста пациента 2

Пациент 3

Мальчик родился от первых срочных оперативных родов (ягодичное предлежание), с низким ростом и весом при рождении, оценкой по шкале Апгар 6/7 баллов (см. табл. 1). При рождении отмечались стигмы дизэмбриогенеза (низко посаженные уши, длинный фильтр, широкий рот), полуптоз (рис. 5а). После рождения наблюдался неврологом с диагнозом: синдром мышечной гипотонии, вентрикуломегалия. У ребенка с раннего возраста отмечались низкие темпы роста, задержка психомоторного и речевого развития. В возрасте 1 года было проведено кариотипирование, по данным которого выявлена делеция короткого плеча 18 хромосомы (46 XY.del(18)(p11.2)). Эпизодов гипогликемий не зафиксировано.

Впервые осмотрен эндокринологом в 2 года, отмечалась выраженная задержка роста (72,3 см, SDS роста: -4,17), в гормональном профиле — сниженный уровень ИФР-1, дефицита других тропных гормонов аденогипофиза выявлено не было (см. табл. 2). Костный возраст в 2 года соответствовал 3 мес. При осмотре отмечалась аномалия развития верхней челюсти (единственный медиальный резец).

По данным МРТ головного мозга в возрасте 2 лет были выявлены характерная для гипопитуитаризма «триада» признаков (гипоплазия аденогипофиза и воронки, эктопия нейрогипофиза), а также перивентрикулярная лейкомаляция, вентрикуломегалия. Ребенку был установлен диагноз: изолированный СТГ-дефицит в составе хромосомной патологии, инициирована терапия рекомбинантным гормоном роста в дозировке 0,025 мг/кг/сут. На момент последнего визита мальчику 2 года 10 мес (рис. 5б), сохраняется задержка речевого развития, наблюдается у логопеда, невролога (см. табл. 3). На фоне лечения отмечалось ускорение темпов роста (рис. 6).

**Figure:**
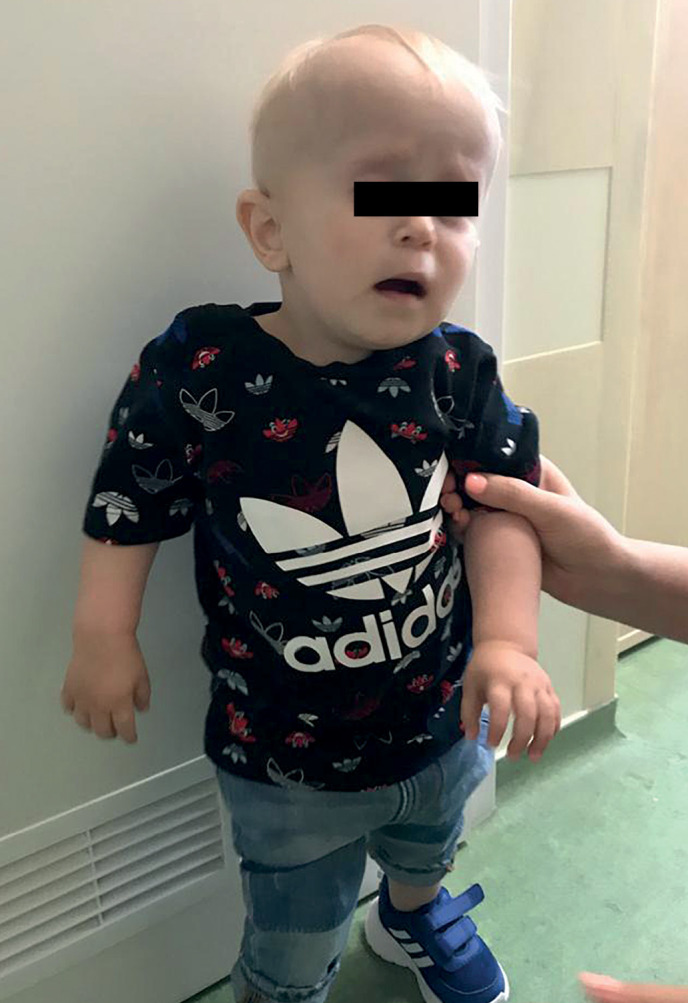
Рисунок 5а. Фото пациента 3 в возрасте 2 года 3 месяца.

**Figure:**
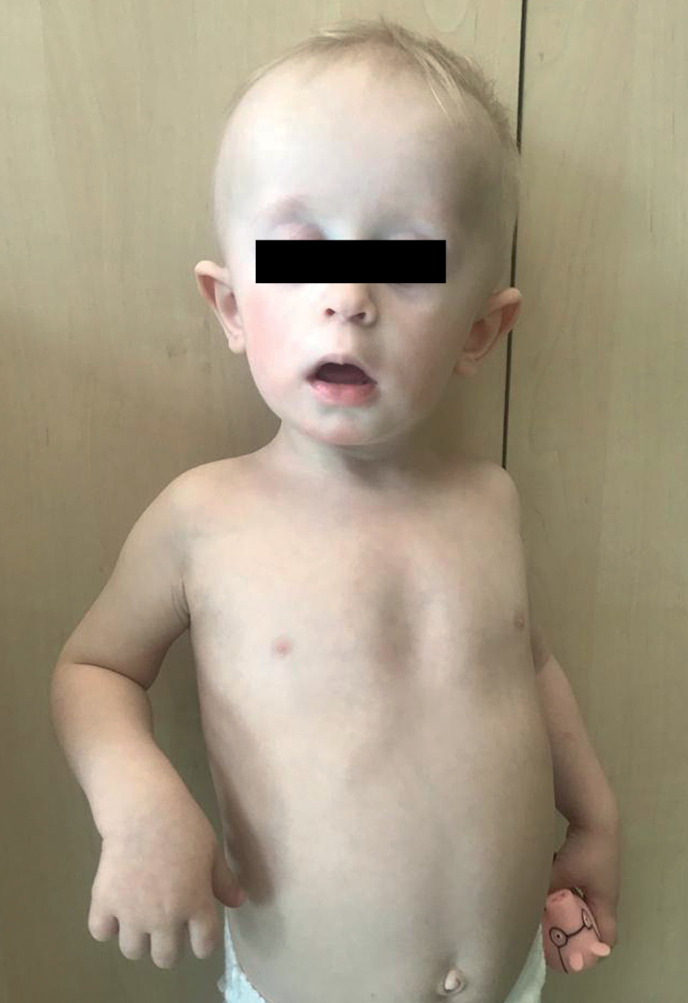
Рисунок 5б. Фото пациента 3 в возрасте 2 года 8 месяцев.

**Figure:**
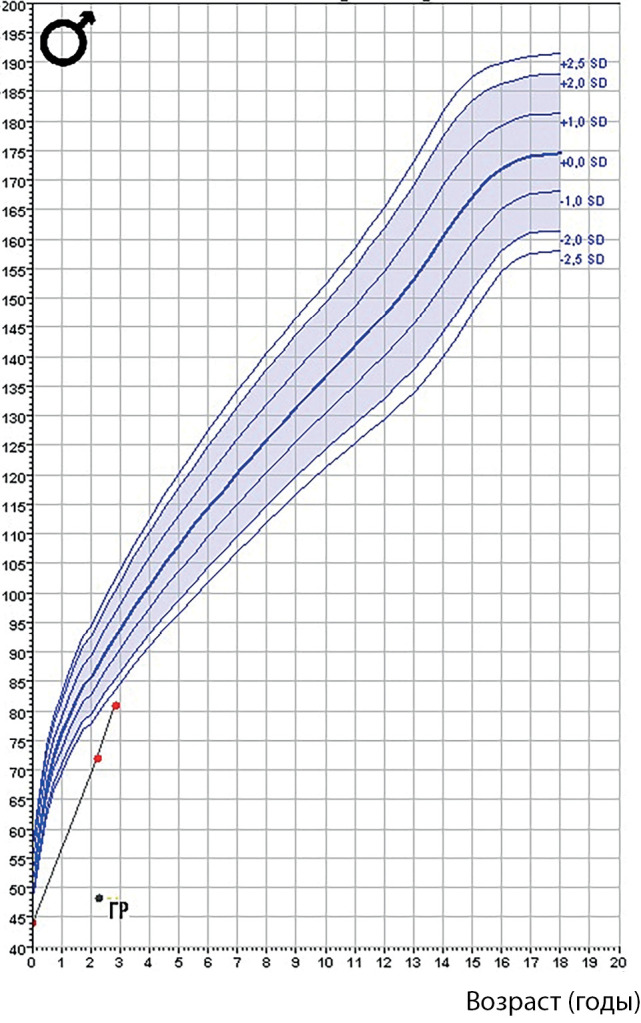
Рисунок 6. График роста пациента 3.

Пациент 4

Девочка родилась от 1-й беременности, срочных родов. При рождении рост и вес соответствовали сроку гестации (см. табл. 1), оценка по шкале Апгар 8/9 баллов. В возрасте 16 ч жизни переведена в отделение реанимации и интенсивной терапии в связи с дыхательными нарушениями (внутриутробная пневмония). В неонатальном периоде отмечалась гипербилирубинемия, эпизодов гипогликемий не зафиксировано. Обращали на себя внимания фенотипические особенности: брахицефальная форма головы, широкая переносица, короткий нос, гипоплазия средней части лица, узкая верхняя губа, опущенные вниз уголки губ, низкорасположенные диспластичные уши, короткая шея, сосковый гипертелоризм, пупочная грыжа, частичная синдактилия 2–3 пальцев стоп, клинодактилия 5 пальцев стоп. С первых месяцев жизни обращали на себя внимание мышечная гипотония, задержка психомоторного развития (голову держит с 4 мес, переворачивается с 6 мес), по поводу которых наблюдалась неврологом и генетиком. В возрасте 8 мес по результатам микроматричного анализа ДНК была диагностирована частичная моносомия по длинному плечу 18 хромосомы (дистальная делеция — 18q22-q23), в связи с чем пациентка была направлена на консультацию к эндокринологу. По данным обследования в гормональном профиле отклонений не было выявлено, отмечалось умеренное снижение ИФР -1 (см. табл. 2). Костный возраст соответствовал паспортному.

В возрасте 1 год 4 мес, несмотря на реабилитационные мероприятия, сохранялась мышечная гипотония, в связи с чем было принято решение об инициации терапии гормоном роста с метаболической целью (0,01 далее 0,025 мг/кг/сут) по решению консилиума. Стимуляционные пробы не проводились ввиду маленького возраста ребенка. На фоне лечения гормоном роста отмечалось нарастание одышки, терапия была прервана на 3 мес. После отмены симптомы дыхательных нарушений исчезли, ребенок был консультирован торакальным хирургом, выявлено неправильное стояние диафрагмального купола по данным рентгенографии. При динамическом наблюдении в возрасте 3 лет отмечалось снижение темпов роста (SDS роста=-2,13). Было выявлено снижение уровня ИФР-1 до 49,8 нг/мл, и терапия гормоном роста была возобновлена в метаболической дозе с постепенным наращиванием до 0,033 мг/кг/сут, ввиду высокой вероятности СТГ-дефицита. При оценке темпов роста на фоне лечения наблюдалась положительная динамика (рис. 7). Побочных эффектов от терапии гормоном роста в дальнейшем не отмечено.

**Figure:**
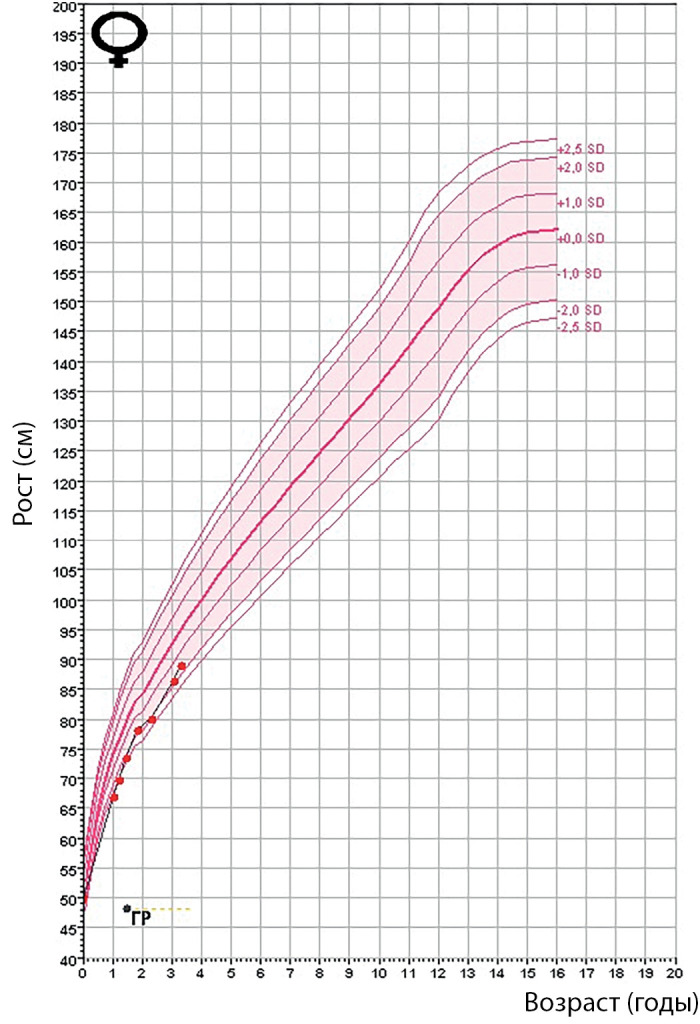
Рисунок 7. График роста пациента 4.

В возрасте 3 лет 3 мес при очередном обследовании выявлена кондуктивная тугоухость.

На фоне активных реабилитационных мероприятий, занятий с логопедом отмечается прогрессия в речевом и психомоторном развитии (см. табл. 3).

## ОБСУЖДЕНИЕ

У троих обследованных нами пациентов с делециями 18 хромосомы имеет место врожденный гипопитуитаризм различной степени выраженности, у одного пациента диагноз носил высоковероятностный характер (Пациент 4). Обращают на себя внимание схожие фенотипические особенности: низкопосаженные диспластичные ушные раковины, короткая шея, узкая верхняя губа, опущенные вниз уголки губ, птоз. В раннем неонатальном периоде у всех детей отмечались мышечная гипотония, дыхательные нарушения, у двух пациентов фиксировалась гипогликемия (Пациенты 1, 2). У всех детей отмечается задержка психомоторного и речевого развития.

У троих пациентов (Пациенты 1, 2, 3) выявлена делеция короткого плеча 18 хромосомы. При этом у Пациентки 1 наиболее крупная делеция (46 ХХ, del (18)(p 11.1; p 11.32)), приведшая к целому спектру врожденных пороков развития (врожденный порок сердца, врожденная катаракта, птоз, множественный дефицит тропных гормонов аденогипофиза). Грубая задержка психомоторного развития у данной пациентки может быть связана как с наличием самого синдрома, так и последствием тяжелых гипогликемий и ишемического поражения ЦНС в неонатальном периоде. У Пациента 2 также была выявлена крупная делеция, сопровождающаяся развитием гипопитуитаризма (см. табл. 1), однако врожденных пороков развития внутренних органов у ребенка не было, в то же время отмечалась гипоплазия зрительных нервов. В соответствии с диагностическими критериями, СТГ-дефицит у данного пациента не был подтвержден [10]. Однако, учитывая наличие рецидивирующих гипогликемий у ребенка грудного возраста с компенсированным вторичным гипокортицизмом, по решению консилиума была назначена заместительная терапия гормоном роста ввиду высокой вероятности наличия СТГ-дефицита. Нейрофиброматоз 2 типа у этого ребенка являлся самостоятельным заболеванием, связанным с мутацией гена NF-2, расположенного на длинном плече 22 хромосомы.

У Пациента 3 с делецией короткого плеча 18 хромосомы отмечается изолированный СТГ-дефицит в сочетании с патологией верхней челюсти (единственный медиальный резец) и птозом.

У всех наших пациентов с делецией короткого плеча (пациенты 1, 2, 3) выявлены структурные аномалии хиазмально-селлярной области, встречающиеся при врожденном гипопитуитаризме (табл. 1).

Короткое плечо 18 хромосомы содержит 66 генов, 12 из которых предположительно являются дозозависимыми [5].

По данным исследований (Cody и соавт., 2009), в области короткого плеча определены регионы, удаление которых приводит к развитию следующих патологий: нейросенсорная и кондуктивная тугоухость (8 и 22% соответственно), косоглазие (38%), птоз (47%), ортопедические проблемы (47%), нистагм (9%), структурные изменения ЦНС по данным МРТ (66%), голопрозэнцефалия (13%), аномалии почек (14%), пороки развития сердца (56%), судороги (13%), врожденная катаракта (6%), задержка речевого развития (100%) [7].

Кроме этого, выделены отдельные гены, гемизиготность которых может приводить к развитию тех или иных состояний. Среди этих генов наиболее хорошо изучены TGIF1, AFG3L2, LAMA1, GNAL, DLGAP1, LCCR30, ANKRD12, IMPA2.

Ген TGIF1 кодирует гомеодоменный белок TWSG, играющий важную роль в сигнальном пути TGF. Мутации гена описаны при развитии голопрозэнцефалии и патологии челюстно-лицевой области. По данным исследований, в 11% у детей с 18p- определяются пороки развития ЦНС голопрозэнцефалического спектра, включая патологию хиазмально-селлярной области. Кроме этого, есть данные, что наличие единственного центрального резца и развитие кондуктивной тугоухости также связано с потерей TGIF1 [11].

Ген AFG3L2 кодирует субъединицу митохондриальных протеаз, участвующих в протеолизе неправильно свернутых белков. Точечные мутации данного гена описаны при спиноцеребральной атаксии 28 типа, для которой характерно развитие атаксии в детском возрасте, дизартрии, нистагма и птоза. Считается, что данный ген обладает неполной пенетрантностью, т.к. его дупликации и делеции описаны в общей популяции. Тем не менее необходимо наблюдение за данной когортой пациентов, учитывая, что заболевание может проявляться в более позднем возрасте и есть сообщение о том, что полная делеция данного гена приводит к фенотипическим проявлениям, как и при точечных мутациях [12].

Ген LAMA1 кодирует белок, участвующий в формировании базальной мембраны. Точечные гомозиготные и компаундные гетерозиготные мутации данного гена описаны при синдроме Poretti–Bolshauser (OMIM#615960), для которого характерны пороки развития мозжечка, миопия, дистрофия сетчатки, нарушение глазодвигательной активности, а также задержка речевого и психомоторного развития.

Ген GNAL кодирует G-альфа — субъединицу рецептора G-белка. Мутации в данном гене описаны при различных формах семейной дистонии. По данным исследований, у 3% пациентов с делецией 18p-, захватывающей область данного гена, была выявлена торсионная дистония.

В области короткого плеча также расположено несколько генов-кандидатов развития аутизма (DLGAP1, LCCR30, ANKRD12, IMPA2), делеции которых, вероятно, можно связать с наличием задержки речевого развития у наших пациентов [5].

Пациенту 3 был выполнен микроматричный хромосомный анализ, по результатам которого удалось выявить гены, попавшие в область делеции. Среди них можно выделить гены AFG3L2, GNAL, LAMA1, TGIF1, удаление которых определяет фенотипические проявления у ребенка (см. табл 1, рис. 5). Требуется более тщательное наблюдение пациента в отношении возможного развития дистонии в будущем.

Таким образом, клинический спектр проявлений синдрома 18p- крайне вариабелен и зависит от размера делеции. В нашем случае ни у одного из пациентов не было потери целого плеча с захватом центромерной области, а имели место частичные делеции различного размера, что объясняет гетерогенность клинических проявлений.

У одного из пациентов (Пациент 4) отмечалась дистальная делеция длинного плеча 18 хромосомы, сопровождающаяся наличием кондуктивной тугоухости, задержкой психомоторного и речевого развития.

Cody и соавт. выделяют 2 области в длинном плече 18 хромосомы: проксимальную (между позицией 19 667 062 и 45 578 734) и дистальную (между 46 739 965 и теломерой), которые имеют клинически значимые отличия. Из 196 генов, локализованных на длинном плече 18 хромосомы, в настоящий момент описано 15, гемизиготность которых клинически значима, но с различной пенетрантностью, таким образом, фенотипические проявления могут значительно варьировать [9].

Путем микроматричного хромосомного анализа у Пациента 4 была точно установлена локализация делеции, что позволило определить гены, выпавшие при данном дефекте (см. табл. 1). Среди них можно выделить гены ZNF236, SALL3, TXNL4A, участвующие в эмбриональном развитии челюстно-лицевой области и формировании слуховых проходов, что может объяснять характерный фенотип.

В настоящее время в дистальном отделе длинного плеча 18 хромосомы не идентифицирован конкретный ген, ответственный за развитие СТГ-дефицита, однако, по данным исследований, именно СТГ-дефицит является патогномоничным для дистального типа делеций 18q и встречается до 56% случаев при данном генетическом нарушении [9][13].

У Пациента 4 не проводились стимуляционные тесты для диагностики СТГ-дефицита ввиду маленького возраста ребенка. Терапия гормоном роста была изначально назначена с метаболической целью, а в дальнейшем — с ростстимулирующей, ввиду снижения SDS роста и снижения уровня ИФР-1. Таким образом, СТГ-дефицит не был доказан лабораторно, однако наличие дистальной делеции длинного плеча 18 хромосомы в сочетании со снижением скорости роста свидетельствует в пользу данного диагноза.

В дистальной части длинного плеча 18 хромосомы выявлены критические регионы для развития врожденных пороков сердца (29%), ортопедической патологии (29%), аутоиммунных заболеваний щитовидной железы (15%), косоглазия (40%), дефицита IgA (18%), а также нарушения миелинизации (97%), не приводящих к прогрессирующим дегенеративным заболеваниям ЦНС [9][14].

У всех детей с данной патологией имеет место задержка интеллектуального и психомоторного развития. По данным исследований было выявлено, что если делеция захватывает область, включающую ген TCF4, то отмечается грубая задержка психомоторного развития. При сохранности гена TCF4 уровень интеллектуального развития в данной группе пациентов варьирует от нормального до средней степени тяжести [15]. У нашего пациента область делеции не захватывала ген TCF4; степень задержки развития оценивается как умеренная, у пациента отмечается прогресс психомоторного развития на фоне реабилитационных мероприятий.

## ЗАКЛЮЧЕНИЕ

Представленные нами клинические случаи демонстрируют фенотипическое разнообразие синдрома 18р- и 18q-. Общими для всех наших пациентов признаками явились наличие дефицита гормона роста, дисморфия лица и задержка речевого развития.

Врожденный гипопитуитаризм представляет собой большую опасность, особенно в неонатальном периоде, так как дефицит кортизола, равно как и дефицит гормона роста, могут приводить к развитию тяжелых рецидивирующих гипогликемий у детей в первые дни и месяцы жизни. Наличие гипогликемического синдрома в период новорожденности в сочетании с холестазом является абсолютным показанием для проведения исследования тропных гормонов гипофиза, позволяющего в максимально ранние сроки верифицировать диагноз и своевременно начать заместительную гормональную терапию.

Врожденный гипопитуитаризм может иметь различную этиологию. Наличие стигм дисэмбриогенеза у детей с полным или частичным выпадением тропных гормонов гипофиза должно служить поводом к проведению кариотипирования или хромосомного микроматричного анализа для исключения патологии 18 хромосомы.

Несмотря на вариабельность клинических проявлений и их степень тяжести у пациентов с делециями 18 хромосомы, точная локализация области дефекта позволяет прогнозировать спектр возможных нарушений и оптимизировать план обследования и наблюдения за такими детьми.

## References

[cit1] de Grouchy J, Lamy M, Thieffry S, et al. Dysmorphie complexe avec oligophrenie: Deletion des bras courts d’un chromosome 17-18. C R Acad Sci. 1963;258:1028.

[cit2] Cody Jannine D., Sebold Courtney, Malik Amtul, Heard Patricia, Carter Erika, Crandall AnaLisa, Soileau Bridgette, Semrud-Clikeman Margaret, Cody Catherine M., Hardies L. Jean, Li Jinqi, Lancaster Jack, Fox Peter T., Stratton Robert F., Perry Brian, Hale Daniel E. (2007). Recurrent interstitial deletions of proximal 18q: A new syndrome involving expressive speech delay. American Journal of Medical Genetics Part A.

[cit3] Cody Jannine D., Hasi Minire, Soileau Bridgette, Heard Patricia, Carter Erika, Sebold Courtney, O’Donnell Louise, Perry Brian, Stratton Robert F., Hale Daniel E. (2013). Establishing a reference group for distal 18q-: clinical description and molecular basis. Human Genetics.

[cit4] Turleau Catherine (2008). Monosomy 18p. Orphanet Journal of Rare Diseases.

[cit5] Hasi-Zogaj Minire, Sebold Courtney, Heard Patricia, Carter Erika, Soileau Bridgette, Hill Annice, Rupert David, Perry Brian, Atkinson Sidney, O'Donnell Louise, Gelfond Jon, Lancaster Jack, Fox Peter T., Hale Daniel E., Cody Jannine D. (2015). A review of 18p deletions. American Journal of Medical Genetics Part C: Seminars in Medical Genetics.

[cit6] Cody Jannine D., Hasi Minire, Soileau Bridgette, Heard Patricia, Carter Erika, Sebold Courtney, O’Donnell Louise, Perry Brian, Stratton Robert F., Hale Daniel E. (2013). Establishing a reference group for distal 18q-: clinical description and molecular basis. Human Genetics.

[cit7] Cody Jannine D., Heard Patricia L., Crandall AnaLisa C., Carter Erika M., Li John, Hardies L. Jean, Lancaster Jack, Perry Brian, Stratton Robert F., Sebold Courtney, Schaub Rebecca L., Soileau Bridgette, Hill Annice, Hasi Minire, Fox Peter T., Hale Daniel E. (2009). Narrowing critical regions and determining penetrance for selected 18q- phenotypes. American Journal of Medical Genetics Part A.

[cit8] Daviss William B., O'Donnell Louise, Soileau Bridgette T., Heard Patricia, Carter Erika, Pliszka Steven R., Gelfond Jonathan A. L., Hale Daniel E., Cody Jannine D. (2013). Mood disorders in individuals with distal 18q deletions. American Journal of Medical Genetics Part B: Neuropsychiatric Genetics.

[cit9] Cody Jannine D., Sebold Courtney, Heard Patricia, Carter Erika, Soileau Bridgette, Hasi-Zogaj Minire, Hill Annice, Rupert David, Perry Brian, O'Donnell Louise, Gelfond Jon, Lancaster Jack, Fox Peter T., Hale Daniel E. (2015). Consequences of chromsome18q deletions. American Journal of Medical Genetics Part C: Seminars in Medical Genetics.

[cit10] Nagaeva Elena V., Shiryaeva Tatiana Y., Peterkova Valentina A., Bezlepkina Olga B., Tiulpakov Anatoly N., Strebkova N. A., Kiiaev Alexey V., Petryaykina Elena E., Bashnina Elena B., Мalievsky Oleg A., Тaranushenko Тatyana Е., Коstrova Irina B., Shapkina Lyubov A., Dedov Ivan I. (2019). Russian national consensus. Diagnostics and treatment of hypopituitarism in children and adolescences. Problems of Endocrinology.

[cit11] Tateossian Hilda, Morse Susan, Parker Andrew, Mburu Philomena, Warr Nick, Acevedo-Arozena Abraham, Cheeseman Michael, Wells Sara, Brown Steve D.M. (2013). Otitis media in the Tgif knockout mouse implicates TGFβ signalling in chronic middle ear inflammatory disease. Human Molecular Genetics.

[cit12] Myers Kenneth A., Warman Chardon Jodi, Huang Lijia, Boycott Kym M. (2014). Deletion ofAFG3L2associated with spinocerebellar ataxia type 28 in the context of multiple genomic anomalies. American Journal of Medical Genetics Part A.

[cit13] Cody Jannine D., Semrud-Clikeman Margaret, Hardies L. Jean, Lancaster Jack, Ghidoni Patricia D., Schaub Rebecca L., Thompson Nora M., Wells Lynda, Cornell John E., Love Tanzy M., Fox Peter T., Leach Robin J., Kaye Celia I., Hale Daniel E. (2005). Growth hormone benefits children with 18q deletions. American Journal of Medical Genetics Part A.

[cit14] Daviss William B., O'Donnell Louise, Soileau Bridgette T., Heard Patricia, Carter Erika, Pliszka Steven R., Gelfond Jonathan A. L., Hale Daniel E., Cody Jannine D. (2013). Mood disorders in individuals with distal 18q deletions. American Journal of Medical Genetics Part B: Neuropsychiatric Genetics.

[cit15] Hasi Minire, Soileau Bridgette, Sebold Courtney, Hill Annice, Hale Daniel E., O’Donnell Louise, Cody Jannine D. (2011). The role of the TCF4 gene in the phenotype of individuals with 18q segmental deletions. Human Genetics.

